# Employee perception of corporate social responsibility authenticity: A multilevel approach

**DOI:** 10.3389/fpsyg.2022.948363

**Published:** 2022-07-26

**Authors:** Hyunok Kim, Myeongju Lee

**Affiliations:** ^1^Department of Business Administration, Kumoh National Institute of Technology, Gumi, South Korea; ^2^Department of Business Administration, Gyeongsang National University, Jinju, South Korea

**Keywords:** corporate social responsibility, CSR authenticity, employee perception, multilevel approach, organizational commitment, construal level theory

## Abstract

Stakeholder interest in the accuracy of Environment Social and Governance (ESG) data and Corporate Social Responsibility (CSR) authenticity has increased, as more companies are disclosing their ESG data. Employees are one of the most important stakeholders of a company, and they have access to more CSR information than other external stakeholders. Employees have a dual role of observing and participating in CSR. Employee perceptions of CSR authenticity play a key role in the positive effects of CSR. In this study, the research model was analyzed through multilevel analysis to contribute to the literature on the mechanism by which CSR affects employees’ job attitudes and perceptions of CSR authenticity. First, hypothesis testing confirmed that external CSR is positively associated with employees’ perceptions of CSR authenticity. Second, CSR authenticity mediates a positive relationship between external CSR and emotional commitment. As the direct effect of external CSR on emotional commitment was not statistically significant, it could be confirmed that the full mediation relationship was significant through CSR authenticity. This study makes three theoretical contributions to the literature on employees’ perceptions of CSR. First, it examines the mechanism of the impact of CSR on employees. By examining the mechanism by which employees recognize and interpret CSR, this study attempts to uncover the black box that CSR affects employees. Second, this study contributes to the literature on CSR authenticity by explaining the mediating role of CSR authenticity in the relationship between CSR and employee job attitudes through construal level theory. Finally, this study contributes to the employee-based CSR literature by analyzing the effect of CSR as an organizational-level variable on emotional commitment as an individual-level variable through multilevel structural equation modeling (MSEM).

## Introduction

As the perception of the necessity of corporate social responsibility spreads, interest in corporate social responsibility activities increases among stakeholders, such as consumers, local communities, government, employees, and shareholders. CSR activities are becoming increasingly important in companies, and empirical research has been actively conducted to verify the positive effects of CSR on companies’ financial performance. In an empirical study of S&P 500 companies, Waddock and Graves (1997) showed that CSR is positively associated with financial performance, such as Return on Equity (ROE), Return on Assets (ROA), and return on sales (ROS). Orlitzky et al. (2003) conducted a meta-analysis of 52 studies and asserted that CSR is positively related to corporate financial performance. In addition, Orlitzky and Benjamin (2001) showed that, as a meta-analysis result, CSR has a positive effect on a company’s financial performance and can also reduce company risk. Moreover, with COVID-19, many companies face problems such as economic recession and developing new business models. These changes have impacted CSR activities, and the need for companies to communicate and report on CSR activities with stakeholders and invest more in stakeholder relationships has become an essential element from a strategic point of view (Ionescu, 2021; Vătămănescu et al., 2021).

Until now, CSR research has mainly focused on firm-level results (Aguinis and Glavas, 2012). Moreover, most studies examine CSR activities and corporate financial performance (Waddock and Graves, 1997; Orlitzky and Benjamin, 2001).

Research on the effects of CSR activities on employees’ attitudes and behaviors have increased in recent years (Aguinis and Glavas, 2012; De Roeck and Maon, 2018). However, it can be seen that there is still a lack of research on how employees participating in CSR activities perceive CSR and what kind of changes they have in their job attitudes according to this perception. Aguinis and Glavas (2012) analyzed CSR studies from 1970 to 2011 based on 588 CSR-related studies and 102 books. As a result, although many studies have been conducted at the macro level on the impact of CSR on the financial and non-financial performance of the company, studies at the micro level on the impact of CSR on employees are still lacking. Since the publication of the study by Aguinis and Glavas (2012), the number of CSR studies, which accounted for approximately 4% of all CSR studies, has increased significantly. However, not much research has been done on employee-focused micro-level studies (Rupp and Mallory, 2015).

The employee plays an important role as a stakeholder who has the dual role of an observer who observes the CSR activities of the company and the role of beneficiary at the same time. Therefore, depending on the role played, the mechanisms by which employees perceive CSR differ. To examine these mechanisms, this study focuses on external CSR.

First, this study examines whether external CSR affects employees’ positive job attitudes (Brammer et al., 2007; Hawn and Ioannou, 2016). This approach is intended to understand employees’ perspectives on CSR and contribute to research on employees’ perceptions of CSR and the underlying mechanisms by which CSR influences employees’ positive job attitudes (Rupp and Mallory, 2015).

Second, by explaining the process of external CSR on employees’ CSR authenticity and examining the relationship between CSR authenticity and organizational commitment, this study contributes to the study of CSR authenticity from the employee’s point of view and compensates for the shortcomings.

Third, by confirming how a company’s CSR activities affect employee-related performance through multilevel analysis, this study enriches the multilevel CSR research, which is necessary to understand the employees’ perspective. As individual-level CSR research measures employees’ perceptions of CSR, a multilevel analysis is needed to examine the impact of firm-level CSR on employees. Moreover, unlike other stakeholders, employees are greatly influenced by organizational contextual factors through the social learning process within the organization. Moreover, since they have a beneficiary’s position in internal-CSR activities, employees’ perceptions of CSR activities can be significantly affected by organizational-level factors. Therefore, it is crucial to examine the effect of organizational contextual factors on employee-level CSR through multilevel analysis. In addition, from a statistical point of view, employees’ data have nested properties within the company. The data of employees belonging to the same organization are inevitably highly correlated owing to the influence of organizational contextual factors. Therefore, the assumption of the ordinary least squares (OLS) error term is violated. If the variance occupied by the firm-level variable from the individual-level variable exceeds 0.10, the standard error may be underestimated and the estimation coefficient overestimated (Maas and Hox, 2004).

Therefore, this study analyzes how firm-level CSR activities affect employees’ CSR perceptions through multilevel analysis and examines the effect of organizational contextual factors that affect employees’ perceptions of CSR.

## Theoretical background and hypotheses

### Corporate social responsibility research from the perspective of employees

Employees’ responses to CSR activities are emerging as an essential research topic (Gond et al., 2017). Previous studies have shown that employees are committed to socially responsible organizations (Valentine and Fleischman, 2008; Stites and Michael, 2011). Specifically, CSR research can be classified into three categories based on employee perspective.

First, some studies have shown that individual employee differences affect their perception of CSR. The individual factors that affect the relationship between CSR and employees’ attitudes include the values and moral orientation of employees (Evans and Davis, 2011), fairness perception (Jones, 2010), degree of individualism or collectivism (Hofman and Newman, 2014), age, and gender (Brammer et al., 2007; Valentine and Fleischman, 2008). Evans and Davis (2011) showed that the positive impact of CSR on employees increased as employees with altruistic tendencies increased, and Hofman and Newman (2014) showed that the more employees have collectivist tendencies, the more positive the impact of CSR on organizational commitment.

Second, research has examined managers or supervisors as factors influencing the effect of CSR on employees; the effects of factors such as leaders’ and managers’ perceptions of CSR, leader-member exchange relationships, and managers’ ethical behaviors were explored (Chen and Hung-Baesecke, 2014; Vlachos et al., 2017). Vlachos et al. (2017) argued that managers’ CSR attributions influence employees’ CSR support through employees’ CSR attributions.

Third, organizational contexts are examined as factors affecting the relationship between CSR and employees’ attitudes. Organizational variables include organizational culture, organizational trust, communication, relational social capital, and CSR implementation (Rupp et al., 2006; Suh, 2016).

Fourth, the effect of CSR on employees’ attitudes has been studied. The effects of CSR on employees include an increase in employer attractiveness (Glavas and Piderit, 2009), employee organizational commitment (Brammer et al., 2007), organizational citizenship behavior (Jones, 2010), and improved employee engagement (Rupp et al., 2018). Moreover, based on the social identity theory, CSR increased employees’ organizational identification (De Roeck et al., 2014) job satisfaction (Vlachos et al., 2013), and turnover intention (Jones, 2010). Also, May et al. (2021) verified the relationship between CSR activity and employee green behavior (EGB) for workers in Malaysia and tested the mediating effect of organizational trust and organizational identity in the relationship between these two variables.

### Corporate social responsibility authenticity

According to Carroll and Wheaton (2009), authenticity can be classified into two types. The first is type or genre authenticity, which means that an object belongs to an original type or classification. This classification means a culturally defined classification, and it may be judged differently by different people. If we take a restaurant as an example, a Korean restaurant that is socially and culturally defined and expected is classified as a restaurant that sells traditional Korean food. Therefore, it can be judged that selling Japanese or Chinese food at a Korean restaurant is not sincere.

Type authenticity of CSR can be interpreted as follows within the organization. Socially and culturally defined CSR encompasses the economic, legal, ethical, and discretionary responsibilities of a company. Therefore, all these responsibilities are fulfilled, and it can be judged that the company’s CSR is sincere.

Second, the moral authenticity of CSR is derived from Heidegger’s existentialist philosophy. This type of authenticity is judging authenticity according to the value intrinsic to an object or the moral meaning of a choice. Being authentic means that an organization’s actions and choices are based on values or morals. Therefore, the moral authenticity of CSR is that a company’s choice to implement CSR reflects its moral beliefs or corporate values.

Corporate social responsibility authenticity is defined as the degree to which CSR activity is socially and culturally defined and expected, reflects corporate beliefs, and is motivated by moral motives (Vermeulen and Barkema, 2002). Studies on CSR authenticity include motivation for CSR, authenticity of CSR, and the effects of CSR authenticity. In general, people tend to be more interested in the causes and motives of behavior than in the behavior itself (Gilbert and Malone, 1995). In particular, there is a tendency to adopt a cynical attitude toward the motives behind CSR activities and policies (Lange and Washburn, 2012). When companies engage in CSR activities to solve poverty or social problems, but exploit resources or the environment, CSR activities are often perceived as greenwashing (Lange and Washburn, 2012). In this case, stakeholders may be confused about judging the company’s CSR activities. However, since employees have more information to judge the degree of commitment of a company to CSR, they can determine the sincerity of a company’s CSR activities more objectively than other stakeholders. Therefore, if employees decide that a company’s CSR activities are not sincere, the impact of CSR activities on employees may be reduced (Rupp et al., 2006).

### External CSR and employee perception of CSR authenticity

External CSR refers to activities that help strengthen a company’s reputation and legitimacy through external stakeholders (Brammer et al., 2007). External CSR includes volunteer activities, donations, environmental protection (Brammer et al., 2007; Hameed et al., 2016). Employees play the role of an observer in external CSR (Rupp and Mallory, 2015). Employee perception of external CSR can be explained based on construal level theory.

Construal level theory describes how people search for and interpret information. Cognitive psychology explains that people use cognitive structures such as schemas to collect and interpret information, which can be abstract, concrete, or factual (Brown, 1958). Abstraction is information compression, which is used for efficient information retrieval and encoding (Burgoon et al., 2013). Abstract cognition is comprehensive but lacks detail, and when using abstract cognition, it may not capture the details in the contexts. Accurate perception is a relatively narrow perspective, and it observes information in more detail, but only sees trees and loses sight of the forest that the trees make up. According to construal level theory, when people interpret and judge an event, a high-level construal or a low-level construal is applied depending on psychological distance. Recognizing that the psychological distance is close, a low-level construal mechanism works (Trope and Liberman, 2010).

Lammers (2012) experimentally examined how people behave differently when using a higher-level construal mechanism and a lower-level construal mechanism. People think abstractly when higher-level construal mechanism is at work, by taking a more holistic view of behavior and characteristics, focus on higher-level goals and events and try to determine what caused the behavior or event. In addition, the idealistic self is emphasized by focusing on internal and ideal values. Thus, more altruistic behaviors were observed. On the other hand, when low-level construal mechanism is at work, people focus on what they can directly experience, such as specific actions, goals, events, and prioritizing actions that benefit them (Kivetz and Tyler, 2007).

Based on construal level theory, employee CSR sensemaking can be applied. When employees perceive CSR, different construal levels can be applied to external and internal CSR. Employees have an observer’s position in external CSR. From the observer’s point of view, external CSR is event-driven by the moral necessity of solving social problems and investing for future generations. It is an event that does not bring immediate benefits to oneself, and the outcome is unpredictable. Therefore, when interpreting CSR as an event that is psychologically distant from external CSR for employees, the higher-level construal mechanism is activated. In the mechanism, an event is observed from an integrated point of view, overall aspect is interpreted, the event’s intention, purpose, and cause is focused upon. Moreover, the ideal self-concept is emphasized by focusing on inner and ideal values (Lammers, 2012).

McShane and Cunningham (2012) studied the factors and results of employee perception of CSR authenticity. When a company engages in CSR activities, it continuously invests the resources. When CSR activities are linked to a company’s products or services, employees perceive that the company’s CSR activities are sincere. In addition, the authenticity of CSR was evaluated by factors such as when an individual employee’s emotional commitment to CSR was high and when he or she perceived the embeddedness of CSR activities.

Beckman et al. (2009) and McShane and Cunningham (2012) showed that resource input and the frequency of CSR activities are factors affecting employee perception of CSR authenticity. Moreover, employees believe that the CSR activities of companies that have invested many resources are sincere. Therefore, it can be predicted that if a company engages in CSR activities, awareness of CSR authenticity will increase.

With this discussion, the following hypothesis was established.

*H1*: External CSR positively affects employees’ perception of CSR authenticity.

### CSR authenticity and organizational commitment

Organizational commitment refers to the identity associated with the organization, wherein the purpose of the organization and individual coincide. Therefore, attitudinal commitment represents the status of an individual’s identification with a specific organization, achieving the organization’s purpose, and staying in the organization (Mowday et al., 1979). CSR affects stakeholders, including employees, who are critical for an organization. Employees can perceive CSR as an expression of organizational values and beliefs, such as an organizational moral orientation. Employee perception of authenticity, fairness, and moral values can positively affect organizational commitment by increasing identification with the organization and emotional attachment (Finegan, 2000).

Meyer and Allen (1991) found that employees seek a positive work environment and experience; if the organization provides such an environment, it encourages employees to continue working in the organization. When employees’ awareness of CSR authenticity increases, they positively perceive the company’s values and beliefs regarding external CSR. Finegan (2000) found that organizational commitment increases by increasing attachment and improving positive job experience. Furthermore, the organizational commitment increases when employees positively perceive the values pursued by the firm. Show that altruistic CSR attribution positively affects employee attitudes such as organizational trust, loyalty, and organizational commitment. On the other hand, when CSR is attributable to selfish motives, these relationships appear negative (Sen et al., 2006; Vlachos et al., 2017).

Therefore, if external CSR increases, the input of resources and the frequency of CSR activities will increase, and employees will judge that the CSR activities of a company that has invested many resources within a limited budget are sincere. When the authenticity of external CSR increases, employees’ ideal self-concept and positive perceptions of corporate values and beliefs, and emotional attachment to the company increases. Employees’ organizational commitment increases as they do not want to leave a company that provides a positive organizational experience.

**Figure 1:**
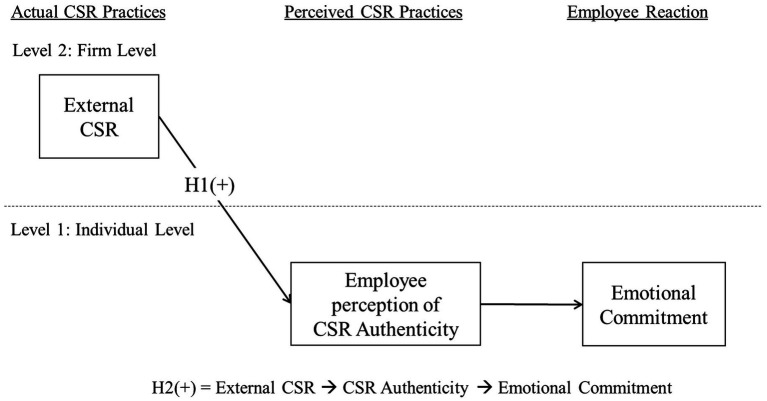
Conceptual model.

With this discussion, the following hypothesis was established. Figure 1 below shows our research model.

*H2*: Employees’ perception of CSR authenticity mediates the positive effect of external CSR on emotional commitment.

## Research design and methods

### Research design

This study examines the effect of firm-level CSR activities on employees’ job attitudes. Therefore, this study was designed as a multilevel analysis. As firm-level data have nested characteristics of employees, the following problems may arise if multilevel analysis is not used when analyzing data. First, if the data are analyzed through simple regression analysis at the individual level, ignoring the influence of firm-level variables, the characteristics of the firm nested in the individual-level data can be ignored. Employees are affected by various variables such as organizational culture and management practices. For this reason, when simple regression analysis is used in nested data, the assumption of the error term in ordinary least squares (OLS) estimation is violated. Second, when individual-level data are aggregated and used at the firm level, the individual characteristics may be ignored. In both cases, the problems of overestimating the regression coefficient and underestimating the standard error may appear (Raudenbush and Bryk, 2002).

Therefore, this study used multilevel analysis to estimate the effect of CSR activities more accurately on employees. Therefore, this study was designed in such a way that data on CSR activities were collected through the company’s CSR manager and employee CSR perception and job attitude were collected from employees.

### Sample and procedures

Although a random sampling method was used to select the study sample for the external validity of the study, the sample was selected in accordance with the following two factors. First, in order to examine the effect of employee perception on CSR activities, which is the purpose of this study, the sample of the study must be a firm that implements a CSR program, and that employees are aware of. Second, in the multilevel analysis, sample selection and size had a significant impact on the research results. An appropriate research sample should be one that can identify differences between firms and simultaneously ensure homogeneity at the level of individuals who share firm characteristics (Klein and Kozlowski, 2000). Therefore, firms suitable for research samples should have implemented CSR programs that are recognized by employees and have differences in firm characteristics such as size, type of industry, listing status, and age of firms. Based on these conditions, data were collected from companies from various industries, sizes, and ages based that have announced CSR activities on their website or issued a white paper for CSR, as surveyed by the Korea Economic Justice Institute (KEJI) Index and the ESG score announced by the Corporate Governance Research Institute.

In multilevel analyses, a clear conclusion has not yet been reached regarding the appropriate number of groups in the sample. Snijders and Bosker (1993) recommended that the appropriate number of groups in a sample should be 20 or more. Maas and Hox (2005) argued that if the number of groups is 30, the standard error is underestimated by 15%; however, if the number of groups increases to 50, the standard error is reduced to less than 5%, which is generally acceptable. In addition, it is also explained that the intraclass correlation coefficient (ICC) value, which represents the proportion of the higher level variance in the total variance, should be at least 0.10(10%).

McNeish and Stapleton (2016) suggested a reasonable number of groups that can be generally accepted in multilevel analysis through simulation analysis in a review of 20 multilevel analysis papers. When the dependent variable is a continuous variable and the full maximum likelihood (FML) estimation method is used, 30 or more groups are recommended, and when the restricted maximum likelihood (REML) estimation method is used, 10 or more groups are recommended. For an accurate estimation of the standard error of variance, it is recommended to secure 50 or more groups. It was suggested that the number of samples in the group should be at least five. Meuleman and Billiet (2009) showed that at least 40 groups are required in the multilevel mediation model, and when the number of groups is 20 or fewer, the error of the estimation coefficient increases dramatically. Therefore, a group sample size of 40 is recommended. Muthén (1991) and Meuleman and Billiet (2009) recommended decreasing the number of respondents in a group and increasing the number of groups. Although there has been some consensus among scholars on the appropriate number of groups in multilevel analysis, the balance between the number of groups and the number of respondents in a group needs further discussion (Preacher et al., 2010). For this reason, this study collected data from more than 50 companies and more than five employees within one company.

Data were collected through a survey. The CSR manager of the company was asked to respond to the firm-level variables, and at least ten employees with various departments and positions within the same company were asked to respond to the employee variables. CSR managers from 65 companies, and 337 employees from 53 companies participated in the survey. The response rate was 85 and 52% for CSR managers and employees, respectively. The average number of employee responses within a company was 6.4. The average value of the respondents was used as the representative value of the company for the data of the five companies in which two or more CSR managers responded. Corporate financial data were collected from annual reports. The characteristics of the firm-level samples can be seen in Table 1 below. Table 2 presents the characteristics of the employee-level samples.

**Table 1:** Data sample characteristics of firms.

		Frequency	Percent (%)	Firm size*	Frequency	Percent (%)
Industry	Manufacturing	25	47.17%	Less than 300	5	9.43%
	Services	11	20.75%			
	Finance and insurance	17	32.08%	300–999	7	13.21%
	Total	53	100%	1,000–1,999	16	30.19%
Listing status	Listed firms	27	50.94%	More than 2,000	25	47.17%
	External audit firms	26	49.06%			
	Total	53	100%	Total	53	100%

^*^Firm size = Number of employees.

**Table 2:** Data sample characteristics of employees.

		Frequency*	Percent (%)
Gender	Male	205	61.93%
	Female	126	38.07%
Education	Graduate from high school	13	3.93%
	Graduate from junior college	24	7.25%
	Bachelor’s degree	262	79.15%
	Master’s and doctorate degrees	32	9.67%
Employment	Permanent employment	320	96.68%
	Temporary employment	11	3.32%
Marital status	Not married	138	41.69%
	Married	193	58.31%
Job level	Nonmanager	260	78.55%
	Manager	71	21.45%
Unionization	Nonunion	127	38.37%
	Refuse to join a union	129	38.97%
	Join a union	75	22.66%
Age	20s	69	20.85%
	30s	164	49.55%
	40s	96	29.00%
	>50s	2	0.60%
Employment period	<1 year	28	8.46%
	1–3 years	48	14.50%
	3 5 years	51	15.41%
	5–10 years	75	22.66%
	More than 10 years	129	38.97%

^*^N = 331.

The manufacturing, financial, and non-financial service sectors were relatively well distributed, and the ratios of listed and unlisted companies were almost the same. However, considering corporate size, due to the nature of CSR programs, most small businesses do not implement CSR, and even if they do, they are implemented only in a specific season in the form of donations. Therefore, they were excluded because employees were not aware of the company’s CSR practices. However, although a branch of a foreign company in Korea usually has a small number of employees, it shows a different aspect from small businesses in CSR activities. It follows the parent company’s CSR policy and implement the same in Korea. Employees were fully aware of this, so small foreign companies were included in the sample of firms with fewer than 300 employees.

As most of the companies in the sample were medium-sized or larger, the majority of the samples were college graduates and regular workers. Notably, marital status and union membership are relatively balanced.

### Measures

#### Corporate social responsibility

In this study, CSR is defined as discretionary corporate activities and policies that meet stakeholder expectations from stakeholder and strategic CSR perspective (Barnett, 2007; De Roeck et al., 2014). There are two ways to measure CSR from the stakeholder’s perspective: objective and subjective measurement. The objective method measures whether the CSR program affects each stakeholder (Hawn and Ioannou, 2016), and the subjective method measures how well a CSR program that affects stakeholders is in place (Turker, 2009; Du et al., 2011). Although objective measurement is good for comparing CSR performance among companies, it is insufficient for reflecting the perception of CSR. This study focuses on employee perceptions of CSR. How CSR is communicated and implemented depends on the perception of CSR managers. Therefore, subjective measures were used in this study (Davies et al., 2001).

Aguinis and Glavas (2019) argue that embedded and peripheral CSR influences employee CSR sensemaking. Embedded CSR means that CSR is integrated into corporate strategy, and that the value of CSR is reflected in the company’s daily work. On the other hand, peripheral CSR means that CSR activities are not related to a company’s business but are treated as secondary activities rather than core activities. If employees perceive CSR to be embedded, it will have a positive effect on employees’ job attitude, but if employees perceive CSR as peripheral, CSR will either not affect or negatively affect employees’ job attitude. When CSR is embedded, employees align the external-oriented CSR with their work at the firm. For example, when a company implements environmental protection for future generations as a core external-oriented CSR activity, employees will be motivated to monitor whether the value of environmental protection is reflected in activities such as procurement of raw materials and manufacture of products. If a company does not care about environmental damage in the production process, employees perceive that the company’s CSR is not sincere. As most of these judgment processes are based on external stakeholders’ CSR, external-oriented CSR affects employees’ sensemaking (Grant, 2007; Aguinis and Glavas, 2019). Therefore, in this study, CSR was measured as external-oriented CSR.

Turker (2009), Du et al. (2011), and Hawn and Ioannou (2016) measured CSR by dividing it into external-oriented CSR and internal-oriented CSR. Turker (2009) measured CSR as a survey method rather than as an objective measurement in the form of a CSR index, and it is the most commonly used measurement among survey methods. Turker (2009) measured CSR for external stakeholders, such as local communities, the environment, future generations, NGOs, customers, and the government. CSR was measured using 12 items adapted from Turker’s external CSR scale. A few sample items are “Our company participates in activities which aim to protect and improve the quality of the natural environment.,” “Our company respects consumer rights beyond the legal requirements.,” “Our company always pays its taxes on a regular and continuing basis,” 7-point Likert scale was used.

#### Corporate social responsibility authenticity

Based on the previous discussion, CSR authenticity was defined into two types. Type authenticity can be interpreted as whether CSR is being implemented in a socially and culturally expected way. Moral authenticity can be explained as to whether CSR is carried out based on a company’s moral beliefs or core values. Thus, CSR authenticity is defined in terms of the perception that the level of CSR activity is socially and culturally defined and expected, reflects corporate beliefs, and is motivated by moral motives (Carroll and Wheaton, 2009). CSR authenticity perception can be interpreted as employees’ perceptions of CSR authenticity. Study by Schaefer and Pettijohn (2006) and Alhouti et al. (2016) were used to modify authenticity perception. CSR authenticity was measured with nine items, such as “Our company’s CSR actions are genuine,” “Our company is standing up for what it believes in,” and “Our company disguises the company’s true thoughts and beliefs in CSR activities” on a 7-point Likert scale. Employees answered CSR authenticity perception questionnaires.

#### Emotional commitment

Emotional commitment was measured using Allen and Meyer’s (1990) multidimensional construct. The most widely used measurement tools related to organizational commitment are Mowday et al.’s (1979) Organizational Commitment Questionnaire (OCQ) and Allen and Meyer’s (1990) multidimensional organizational commitment measurement. Mowday et al. (1979) defined organizational commitment based on psychological attachment and explained it as a psychological state that believes in organizational values and beliefs and is willing to work and engage in the organization. Allen and Meyer (1990) defined organizational commitment as organizational identification, involvement as an instrumental need, attachment to the organization according to individual values, and an attitude of remaining in the organization. This was measured by dividing the scale into three dimensions. As CSR is related to psychological attachment and personal moral beliefs and values, the multidimensional measurement was used to examine the effect of CSR on emotional commitment among the dimensions of organizational commitment. Emotional commitment was measured using eight items and a 7-point Likert scale was used. Sample items were “I would be very happy to spend the rest of my career with this organization,” “I really feel as if this organization’s problems are my own,” and “I do not feel a strong sense of belonging to my organization.”

#### Controls

Control variables were selected as those that can affect CSR at the firm level and those that can affect emotional commitment at the individual level. Industry, previous year’s sales, union presence, listing status, and firm age were controlled as firm characteristics. We thought it was important to explain the relationship between the slack resources and CSR and to consider them as a variable. We included slack resources as a control variable in our analysis. In general, when using slack resources as a control variable, researchers control R&D intensity calculated as a ratio of investment in R&D by total assets, and advertising expenditure (McWilliams and Siegel, 2000; Hawn and Ioannou, 2016). To control other intangibles that may affect the perception of CSR and commitment, we included gender, education, marital status, and age as individual characteristics. We also controlled for employment type, job level, union membership, and employee tenure because it may be related to commitment at the individual level (Waddock and Graves, 1997). Gond et al. (2010) analyzed the antecedent variables, outcome variables, and research results of several studies that focused on the effect of CSR on employees. Numerous studies have used age, gender, tenure, firm size, educational level, and position as control variables (Brammer et al., 2007; Valentine and Fleischman 2008).

### Analytic strategy

A multilevel analysis method was used in this study to examine the effect of firm-level CSR on employee perceptions of CSR and emotional commitment, which are individual-level variables. Multilevel analysis methods can be divided based on hierarchical linear modeling and those based on structural equation modeling. The hierarchical linear modeling (HLM) program developed by Bryk and Raudenbush (1992) is widely used for multilevel analysis based on hierarchical linear modeling. Although this program has the advantage of analyzing the cross-level direct effect and the cross-level interaction effect, it does not examine the complex relationship between variables (Klein and Kozlowski, 2000).

The development of approaches using structural equation models in multilevel analysis has made it possible to estimate multilevel structural equation models in programs such as M-plus (Asparouhov and Muthen, 2008). A multilevel mixture model or multilevel model with latent variables estimates the integrated model by estimating each model between and within levels (Hox and Maas, 2001). Therefore, I analyzed the research model using the M-plus program because it is possible to analyze complex relationships between variables and to estimate between and within levels in one model, respectively.

In multilevel structural equation modeling, when explaining the cross-level direct effect model, assuming that the dependent variable is $Y_{ij}$ , the explanatory variable is $X_{ij}$ , and the group-level explanatory variable is $W_{j}$ , the following formula is used:


(1) $$Y_{ij}=\beta_{0j}+\beta_{1j}X_{ij}+r_{ij},$$

(2) $$\beta_{0j}=\gamma_{00}+\gamma_{01}W_{j}+u_{oj},$$

(3) $$\beta_{1j}=\gamma_{10}+\gamma_{11}W_{j}+u_{1j},$$

(4) $$Y_{ij}=\gamma_{00}+\gamma_{10}X_{ij}+\gamma_{01}W_{j}+\gamma_{11}X_{ij}W_{j}+u_{1j}X_{ij}+u_{0j}+r_{ij}$$

In Equation (4), $\gamma_{10}$ is the effect of $X_{i}$ on $Y_{ij}$ , and $\gamma_{01}$ is the effect of the latent group average, not the observed group average. In this study, when estimating the mediation model, a multilevel model with latent variables was used to estimate complex relationships between variables.

The method used in the estimation of statistical outputs is robust maximum likelihood estimation (MLR) using the expectation–maximization (EM) algorithm. The EM algorithm estimates the sufficient statistics required for parameter estimation as expected values and calculates the maximum likelihood estimate based on it. This approach can be considered an empirical Bayesian estimation method. The Bayesian estimation method uses knowledge of the prior distribution of parameters, but empirical Bayesian estimation uses parameters estimated from observation data for convenience. Therefore, empirical Bayesian estimation can be used without prior knowledge of the distribution of parameters (Kang, 2016). The difference between MLR estimation using the EM algorithm and the commonly used maximum likelihood estimation (ML) is that the ML estimation is sensitive to the number of samples and missing data. However, MLR estimation has the advantage that it is less sensitive to the number of samples and missing data, and is more favorable to the assumption of normality. This is because the MLR estimation estimates the full data sufficient statistic required for parameter estimation as an expected value and calculates the maximum likelihood estimate based on it. Because of the assumption that all regression coefficients have a random effect, MLR estimation takes more time for model estimation, and a coefficient corrected for the chi-square value should be used when evaluating the model fit.

## Results

### Descriptive statistics and correlations

Before hypothesis testing, descriptive statistics and correlations between variables were analyzed. The following Tables 3 and 4 show the descriptive statistics and correlation analysis results for each level. First, in the correlation analysis results of individual-level variables, only job level and age were significantly correlated with CSR authenticity perception. Managers had a higher perception of CSR authenticity than team members (*r* = −0.15, *p* < 0.01), and older employees had a higher perception of CSR authenticity (*r* = 0.13, *p* < 0.05).

**Table 3:** Descriptive statistics and correlations among individual-level variables.

Individual level		Mean	SD	1	2	3	4	5	6	7	8	9	10
1	Gender	0.38	0.49										
2	Age	35.71	6.49	−0.37^**^									
3	Employment	0.97	0.18	−0.13^*^	0.18^**^								
4	Marriage	0.59	0.49	−0.14^**^	0.55^**^	0.12^*^							
5	Job level	0.79	0.41	0.26^**^	−0.58^**^	−0.01	−0.30^**^						
6	Education	2.95	0.57	−0.14^**^	0.20^**^	0.13^*^	0.07	−0.09					
7	Job	2.34	0.60	0.00	0.05	0.08	0.06	0.02	0.05				
8	Union	1.84	0.77	0.08	0.11^*^	0.09	0.10	0.14^**^	0.02	0.06			
9	Tenure	4.81	3.75	−0.05	−0.08	−0.02	−0.13^*^	0.06	0.05	0.02	0.11		
10	Authenticity	4.51	1.16	−0.04	0.13^*^	−0.05	0.06	−0.15^**^	0.02	−0.08	−0.02	0.00	
11	Emotional commitment	4.98	1.13	−0.14^*^	0.29^**^	−0.01	0.16^**^	−0.28^**^	0.07	−0.02	−0.04	−0.05	0.62^**^

N = 331, 1. Gender: 1 = Male, 2 = Female, 3. Employment: 0 = Temporary employment, 1 = Permanent employment, 4. Marriage: 0 = Not married, 1 = Married, 5. Job level: 0 = Manager, 1 = Nonmanager, 6. Education: 1 = High school, 2 = Junior college, 3 = University, 4 = Graduate school, 7. Job: 1 = Production worker, 2 = Management, 3 = Sales work, 4 = research and development (R&D), 8. Union: 1 = Nonunion, 2 = Refuse to join a union, 3 = Join a union.

* *p* < 0.05.

** *p* < 0.01.

**Table 4:** Descriptive statistics and correlations among firm-level variables.

Firm level		Mean	SD	1	2	3	4	5	6	7
1	Industry	1.85	0.89							
2	Listed firms	2.04	0.98	0.21						
3	Firm age	41.49	19.16	−0.23	−0.12					
4	Unionization	1.43	0.50	0.24	0.48^**^	−0.42^**^				
5	Sales	21.03	2.05	−0.06	−0.13	0.44^**^	−0.34^*^			
6	AD intensity	4.62	0.02	0.05	−0.11	−0.03	0.08	−0.23^**^		
7	R&D intensity	4.62	0.02	−0.30^*^	−0.10	0.17	0.02	0.18^**^	0.42^**^	
8	External CSR	5.41	0.95	0.29^*^	0.12	0.27	−0.14	0.32^**^	0.07	0.02

*N* = 53, 1. Industry: 1, Manufacturing; 2, Finance and Insurance; 3, Services. 2. Listed firms: 1 = Korea stock price index (KOSPI), 2 = Korea securities dealers automated quotation (KOSDAQ), 3 = External audit firms, 4. Union: 1, Union; 2, Nonunion, 6.advertising intensity, 7. research and development intensity, 8. External-oriented corporate social responsibility.

* *p* < 0.05.

** *p* < 0.01.

Second, the results of the correlation analysis of firm-level variables show that external-oriented CSR is implemented more in the financial industry than in the manufacturing and service industries (*r* = 0.29, *p* < 0.05), and companies with high sales in the previous year (*r* = 0.32, *p* < 0.01).

### Confirmatory factor analyses

First, confirmatory factor analysis was conducted to evaluate the validity and reliability of the variables (see Table 5). The factor loadings of all the latent variables were significant at *p* < 0.001. Although some factors showed low factor loadings, all other factors loaded significantly and met the recommended cut-off of 0.7. Kline (2011) stated that convergent validity is secured only when the factor loadings meet the cutoff of 0.7 or higher, but it is not a problem if there is sufficient theoretical basis, so the measurement variables presented in the table were used.

**Table 5:** Results of confirmatory factor analysis.

Latent variables		Measurement variables	Standardization^*^	Standard error	Cronbach alpha
Employee perception of CSR authenticity	CSR authenticity1	CSR authenticity 1	0.887	0.014	0.944
		CSR authenticity 2	0.913	0.011	
		CSR authenticity 3	0.901	0.012	
		CSR authenticity 8	0.658	0.033	
		CSR authenticity 9	0.844	0.018	
	CSR authenticity2	CSR authenticity 4	0.879	0.016	
		CSR authenticity 5	0.860	0.018	
		CSR authenticity6	0.830	0.020	
Emotional commitment	EC 1		0.679	0.032	0.895
	EC 2		0.598	0.038	
	EC 5		0.820	0.021	
	EC 6		0.901	0.014	
	EC 7		0.795	0.023	
	EC 8		0.830	0.020	

* All measurement parameters were statistically significant at the level of *p* < 0.001.

Second, the reliability of each measurement variable was evaluated. Cronbach’s alphas of the two latent variables were 0.895 and 0.944, indicating that the reliability of the measured variables was at an appropriate level (Nunnally and Bernstein, 1994). We found acceptable within-group agreement for each construct and significant between-group variances in the individual-level variables (see Table 6).

**Table 6:** Within-group agreement and ICC value of the dependent variable.

	ICC(1)	ICC(2)	$r_{wg}$
Emotional commitment	0.25	0.70	0.85
CSR authenticity	0.30	0.73	0.85

1. Intraclass correlation coefficient (ICC) (1): Proportion of between-group variance in total variance, 2. ICC (2): ICC (2) represents the reliability of the group mean by adjusting the ICC(1) using the group size. 3. $r_{\mathrm{wg}}$ : Within-group agreement.

Finally, the model fit was compared through multilevel confirmatory factor analysis. We estimated a one-factor model in which CSR authenticity and emotional commitment were grouped as one factor. We also estimated a two-factor model, in which our theoretical model, CSR authenticity, and emotional commitment were divided into individual factors. We also estimated a three-factor model, including a higher-order factor model, in which CSR authenticity was divided into two factors (see Table 7).

**Table 7:** Comparison of factor models.

	AIC	BIC	$\chi^{2}$	*df*	$\Delta \chi^{2}$ ( Δdf)	RMSEA	CFI	TLI	SRMR	GFI
One-factor model CA, EC were blended	12,844	13,047	320.62	66		0.11(0.10–0.12)	0.934	0.909	0.076	0.919
Two-factor model	12,699	12,905	173.54	65	147.08(1)^***^	0.07(0.06-0.83)	0.972	0.961	0.036	0.956
Three-factor model CA was separated into two factors	12,679	12,893	149.30	63	24.24(2)^***^	0.06(0.05-0.77)	0.978	0.968	0.033	0.962

1. Akaike information criterion (AIC), 2. Bayes information criterion (BIC), 3. Degree of freedom (DF), 4. Root mean square error of approximation (RMSEA), 5. Comparative fit index (CFI), 6. Tucker–Lewis index (TLI), and 7. Standard root mean square residual (SRMR).

*** *p* < 0.001.

As for the comparative fit index (CFI) value proposed by Bentler (1990), it can be judged that a model with a value of 0.90 or higher has an appropriate fit, a model with a value of 0.95 or higher has a good fit, and the CFI value of the one-factor model is 0.934, which can be evaluated as an appropriate fit. Browne and Cudeck (1993) reported that the root mean square error of approximation (RMSEA) value of 0.05 or less had a good fit, and that between 0.05 and 0.08 had a fair fit. For the one-factor model, 0.07 (90% confidence interval (CI), 0.06–0.08) was evaluated as appropriate. Hu and Bentler (1999) suggested that if the standard root mean square residual (SRMR) value is 0.08 or less, the model fit is appropriate. As the one-factor model had an SRMR value of 0.074, it can be considered that the model fit was appropriate. As such, the absolute model fit of the one-, two-, and three-factor models can be evaluated as appropriate. Therefore, the relative fit of the three models was evaluated based on the parsimonious model. When the models were compared using the chi-square value, the three-factor model with a higher-order factor was found to be the most appropriate model. Therefore, a three-factor model was selected as the final model.

### Hypothesis testing

We first controlled for variables that could affect CSR at the firm level and those that could affect emotional commitment at the individual level. Industry, previous year’s sales, union presence, listing status, advertising intensity, R&D intensity, and firm age were controlled for firm-level variables, while gender, education level, employment type and level, marital status, union membership, age, and tenure were controlled for individual-level variables (Waddock and Graves, 1997; McWilliams and Siegel, 2000; Hawn and Ioannou, 2016).

**Figure 2:**
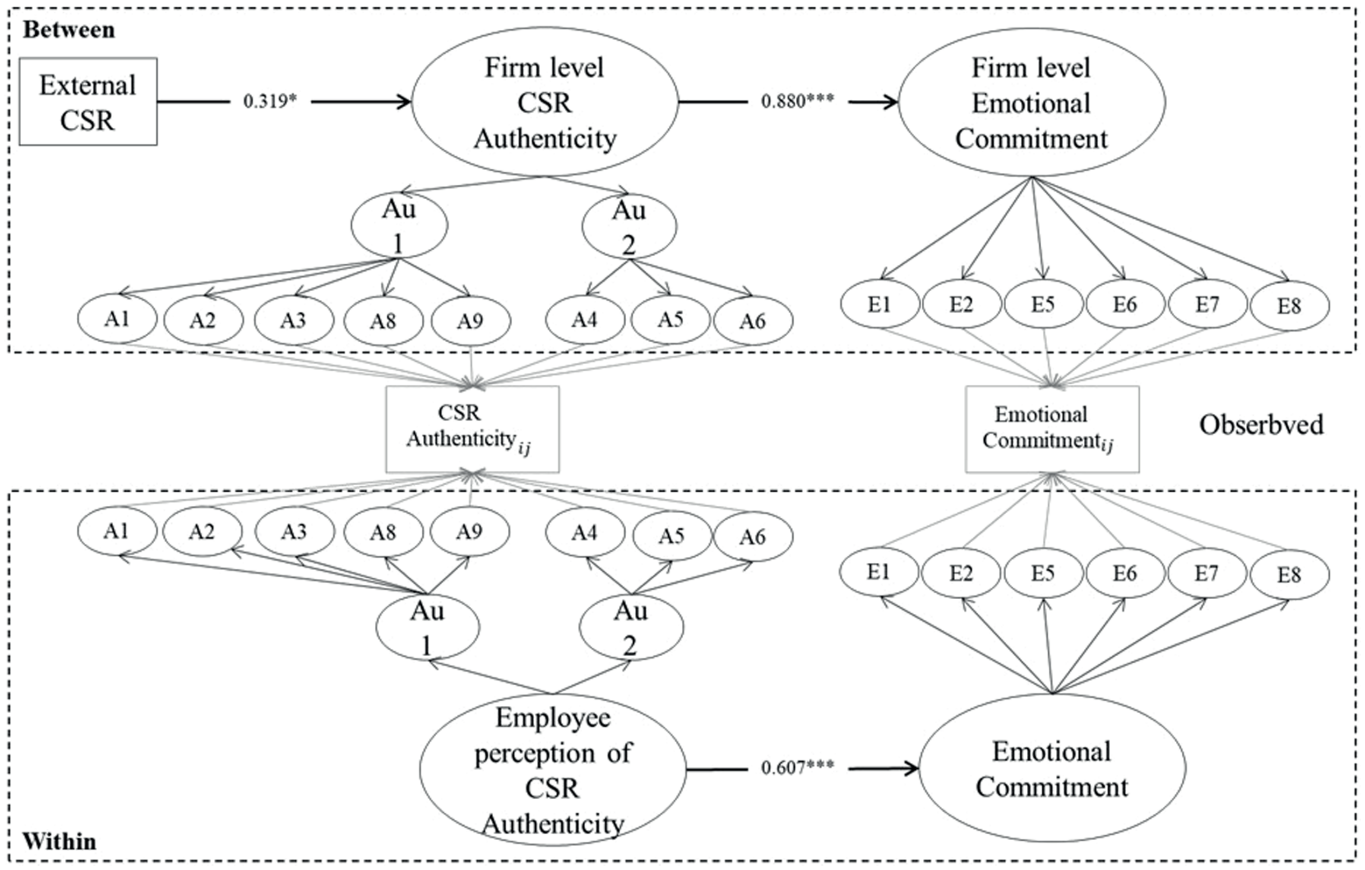
Multilevel SEM test of hypothesized relationships (full mediation). Factor loadings are not displayed Estimates reported are standardized values. We control for industry, listed firms, firm age, unionization, sales, advertisement intensity, and intensity at the firm level, and gender, age, employment, marriage, job level, education, job, union, and tenure at the individual level (not shown) in this model.

We tested our theoretical model using MSEM. As shown in Figure 2, in MSEM, it can be conceptually understood that regression analysis is performed at the group and individual levels. The fit of a model is generally evaluated by comparing the absolute values of the models. This is because it is not appropriate to use CFI, chi-square value, and RMSEA, which are sensitive to the number of samples, as the number of group-level samples is small (Muthén, 1994; Hox, 1995; Ryu, 2014). Therefore, the fit of the model was evaluated using the SRMR value, which measures the fit of the model at the group and individual levels. The statistical power of the estimation results was confirmed through a Monte Carlo simulation. The overall results of the proposed model indicated an acceptable level of model fit (RMSEA = 0.094, SRMR within = 0.068, SRMR between = 0.063).

The results of hypothesis testing are presented in Table 8. Hypothesis 1 predicted that external CSR is positively associated with employees’ perceptions of CSR authenticity. As expected, external CSR is positively related to CSR authenticity ( β= 0.124, *p* < 0.05). Thus, Hypothesis 1 is supported. Hypothesis 2 proposed that firm-level external CSR would significantly and indirectly relate to emotional commitment through employee perception of CSR authenticity. Our findings support hypothesis 2. CSR authenticity and emotional commitment are positively linked at both the individual and the firm-level ( withinβ= 0.604, *p* < 0.001, betweenβ= 0.880, *p* < 0.001). Regarding the effect of CSR authenticity perception on emotional commitment, the firm effect was larger than the individual effect. These results show that the average emotional commitment of employees is positively high in firms with a high level of CSR authenticity.

**Table 8:** Multilevel structural equation modeling results (full mediation model).

Estimates	*B*	Standard error
Within level		
CSR authenticity→Emotional commitment	0.607^***^	0.085
Between level		
CSR→CSR authenticity	0.319^*^	0.134
CSR authenticity→Emotional commitment	0.880^***^	0.130
Cross-level indirect effect		
CSR→CSR authenticity→Emotional commitment	0.281^*^	0.136

Level 2 controls: industry, listed firms, firm age, unionization, sales, advertisement intensity, research and development intensity, Level 1 controls: gender, age, employment, marriage, job level, education, job, union, tenure.

* *p* < 0.05;

** *p* < 0.01;

*** *p* < 0.001.

The proposed mediation model was tested. The direct effect of external CSR on emotional commitment was not statistically significant. We found support for the indirect effect of external CSR on aggregate emotional commitment through CSR authenticity. The coefficient of the indirect effect was 0.281, and the 95% bias-corrected bootstrap CI was (0.013 and 0.548). As zero was not included in the 95% CI, we conclude that CSR authenticity has a robust mediation effect on the relationship between external CSR and emotional commitment. The results showed that external CSR has a positive effect on emotional commitment only through CSR authenticity. In other words, employees’ perceptions of CSR authenticity fully mediate the relationship between external CSR and emotional commitment.

To further test the mediation effect of the proposed model, we examined the alternative model in which the direct path from external CSR to emotional commitment was added. The chi-square difference test indicated that the fit of the alternative model was similar to that of the proposed model ( $\Delta \chi^{2}$ (1) =1.763, *p* = 0.184). Thus, these results provided evidence that the proposed model had a fully mediating effect.

## Discussion

As an increasing number of companies disclose their ESG data, stakeholder interest in the accuracy of ESG data and CSR authenticity has increased. Employees are one of the important stakeholders of a company, and they have access to more CSR information than other external stakeholders. Employees are stakeholders with a dual role of observing and participating in CSR. Therefore, by performing their daily duties in the company, employees can easily recognize the CSR philosophy and values nested in management activities, and through this, they can judge the authenticity of CSR. And employee perception of CSR authenticity can affect the job attitude of employees. Negative perceptions of green washing and CSR authenticity may weaken the positive impact of CSR on job attitudes, or even turn it into a negative impact (Aguinis and Glavas, 2019). Therefore, the relationship between employees CSR sensemaking mechanism and CSR authenticity perception needs to be researched. In this study, the research model was analyzed through multilevel analysis to contribute to the literature on the mechanism by which CSR affects employees’ job attitudes and perceptions of CSR authenticity.

### Research implications

This study makes three theoretical contributions to the literature on employee perception of CSR. First, it examines the mechanism of the impact of CSR on employees. By examining the mechanism by which employees recognize and interpret CSR, this study attempts to uncover the black box of effect of CSR on employees. Based on construal level theory, the effect of external CSR on emotional commitment, an important performance predictor at the employee level, was examined. Hypothesis 1 predicted that external CSR would be positively linked to employees’ perceptions of CSR authenticity. Hypothesis testing confirmed that external CSR is positively associated with employees’ perceptions of CSR authenticity. Hypothesis 2 proposed that external CSR is positively linked with emotional commitment and that employee perception of CSR authenticity mediates the positive relationship between external CSR and emotional commitment. As expected, CSR authenticity mediated the positive relationship between external CSR and emotional commitment. As the direct effect of external CSR on emotional commitment was not statistically significant, it could be confirmed that the full mediation relationship was significant through CSR authenticity.

Second, this study contributes to the literature on CSR authenticity by explaining the mediating role of CSR authenticity in the relationship between CSR and employee job attitudes through construal level theory. Based on the construal level theory, the research results can be explained as follows.

First, what it means for a company to engage in external CSR is to invest its limited resources in external CSR. In this case, employees’ awareness of the company’s CSR authenticity will increase by recognizing that the company is engaged in CSR. Second, external CSR is an activity that targets external stakeholders and is mainly related to moral values or beliefs, such as the promotion of social values, protection of consumer rights, environmental protection, resolution of poverty, and sustainable development for future generations. Based on construal level theory, a higher-level construal mechanism will be applied because the psychological distance between employees and external CSR is far. When a higher-level construal mechanism is applied, employees perceive CSR from integrated and normative perspectives, such as CSR motives, objectives, and moral beliefs (Trope and Liberman, 2010). If so, it can be explained that when external CSR increases, it is perceived as an expression of organizational values and beliefs, such as moral orientation, generosity, and respect, and consequently, employee perceptions of CSR authenticity increase. When employee perception of CSR authenticity increases, they perceive the organization’s values and beliefs positively, resulting in an increase in emotional commitment by increasing employees’ emotional attachment to the organization and improving positive job experience (Finegan, 2000).

Finally, this study contributes to employee-based CSR literature by analyzing the effect of CSR as an organizational-level variable on emotional commitment as an individual-level variable through MSEM. Firm-level variable data were collected from CSR managers and data for individual-level variables were collected from employees of the firm for MSEM. Aguinis and Glavas (2012) reports that only 9 of the 181 (i.e., 5%) studies used multilevel analysis. They argued that there is a knowledge gap about the relationship between higher-level antecedents and lower-level outcomes. This study fills this knowledge gap through multi-level analysis.

### Practical implications

Employees view external CSR from the perspective of authenticity. The study results show that if employees judge the external CSR as not sincere, the external CSR did not have a positive effect on employees’ emotional commitment. CSR managers can use the findings of the current study to establish strategies related to external CSR. When communicating with employees about external CSR, the purpose and intention of CSR, CSR philosophy, and moral beliefs must be communicated from an integrated and global perspective.

Employee perception of CSR authenticity plays a key role in the positive effects of CSR. Although many external CSR activities can have a positive effect on the perception of CSR authenticity, investing many resources in CSR may not always have a positive effect on the perception of CSR authenticity. In addition, if employees do not perceive CSR activities as sincere, the positive impact of CSR on corporate performance is greatly reduced. Therefore, CSR managers must continuously measure and manage employee perception of CSR authenticity.

### Limitations and future research

Efforts have been made to secure the reliability and validity of the study, but this study has several limitations, so it is necessary to supplement these areas in future studies. First, it is necessary to collect more sample groups. In this study, we collected data by meeting the minimum number of group samples that did not affect the estimated results proposed by Snijders and Bosker (1993), Maas and Hox (2005), Preacher et al. (2010), and McNeish and Stapleton (2016). As the number of group samples increased, the effect on standard errors and estimation errors decreased. In future studies, it will be necessary to secure a sufficient number of group samples (100 or more). However, because the researchers’ agreement on the number of respondents in the group is not yet clear, it should be decided based on the results of various simulations studied later.

This study also examined the bias of the research results that may occur due to the lack of group samples by checking the statistical power using Monte Carlo simulations. As a result of checking the statistical power, when the number of group samples was 53, the statistical power was 0.989 or higher, confirming that there was no problem due to the number of group samples in the study results. When the number of group samples was 100, the statistical power was 1.000.

Second, longitudinal studies are needed. In this study, both the predictor variables, CSR and perception of CSR authenticity, were measured at the same time point as the dependent variable. In the case of emotional commitment, the dependent variable, compared to other job attitudes such as job satisfaction, has somewhat stable characteristics over time (Mowday et al., 1979), so it is necessary to observe with a time difference. Therefore, in future research, it is necessary to proceed with the longitudinal research method.

Third, it is necessary to modify the estimation method. In this study, MLR and REML estimation methods were used in the multilevel model. Although this estimation method is less sensitive to the number of samples in the group and provides a more robust estimation result than the general ML estimation method, it has limitations in supplementing the assumption of distribution in a multilevel mediation model (Preacher et al., 2010). An estimation method that has recently attracted attention in multilevel analysis is the empirical Bayesian estimation method. The simulation results were published using the empirical Bayesian estimation method. The estimation method using the Bayesian confidence interval provided more robust estimation results than the maximum likelihood estimation. In future research, it will be necessary to analyze multilevel data through estimation of multilevel structural equation modeling using empirical Bayesian estimation.

Finally, it is necessary to study the various organizational contextual factors that can affect employee perception of CSR authenticity. The perception of CSR authenticity is an important factor in the influence of external CSR on employees. Further research is needed on firm-level variables that can increase perceptions of CSR authenticity. In addition, studies on the interactions between these contextual factors are needed.

## Conclusion

This study aims to uncover the “black box” that CSR influences employees’ positive job attitudes. In particular, we focused on the mechanism of how employees interpret external CSR. According to construal level theory, when employees interpret external CSR, which is psychologically distant, it can be interpreted based on the purpose of the behavior and moral principles. As a result of examining the data, it was found that external CSR affects emotional commitment through CSR authenticity. In other words, CSR authenticity had the effect of fully mediating the relationship between external CSR and emotional commitment. Although there are prior literatures in which employee’s perceived CSR is significantly associated with organizational commitment, there are few studies regarding the relationship between firm-level CSR and organizational commitment (Aguinis and Glavas, 2019). This study contributes to the employee-based CSR research by analyzing this mediating relationship using the firm-level CSR variable based on the MSEM method.

## Data availability statement

The datasets presented in this article are not readily available because restrictions apply to the availability of these data, which were used under the Statistics Act for this study. Data are only available with the permission of the participants. Requests to access the datasets should be directed to hokim0@kumoh.ac.kr.

## Author contributions

This study was based on HK’s doctoral thesis. HK designed the study, analyzed the data, and wrote the manuscript. ML wrote and reviewed the manuscript. All authors have contributed to the manuscript and approved the submitted version.

## Conflict of interest

The authors declare that the research was conducted in the absence of any commercial or financial relationships that could be construed as a potential conflict of interest.

## Publisher’s note

All claims expressed in this article are solely those of the authors and do not necessarily represent those of their affiliated organizations, or those of the publisher, the editors and the reviewers. Any product that may be evaluated in this article, or claim that may be made by its manufacturer, is not guaranteed or endorsed by the publisher.

## References

[ref1] Aguinis H. Glavas A. (2012). What we know and don’t know about corporate social responsibility: a review and research agenda. J. Manag. 38, 932–968. doi: 10.1177/0149206311436079

[ref2] Aguinis H. Glavas A. (2019). On corporate social responsibility, sensemaking, and the search for meaningfulness through work. J. Manag. 45, 1057–1086. doi: 10.1177/0149206317691575

[ref3] Alhouti S. Johnson C. M. Holloway B. B. (2016). Corporate social responsibility authenticity: investigating its antecedents and outcomes. J. Bus. Res. 69, 1242–1249. doi: 10.1016/j.jbusres.2015.09.007

[ref4] Allen N. J. Meyer J. P. (1990). The measurement and antecedents of affective, continuance and normative commitment to the organization. J. Occup. Psychol. 63, 1–18. doi: 10.1111/j.2044-8325.1990.tb00506.x

[ref5] Asparouhov T. Muthen B. (2008). “Multilevel mixture models,” in *Advances in Latent Variable Mixture Models [Internet]*. eds. G. R. Hancock and K. M. Samuelsen (Information Age Publishing, Incorporated) (CILVR series on latent variable methodology).

[ref6] Barnett M. L. (2007). Stakeholder influence capacity and the variability of financial returns to corporate social responsibility. Acad. Manag. Rev. 32, 794–816. doi: 10.5465/amr.2007.25275520

[ref7] Beckman T. Colwell A. Cunningham P. H. (2009). The emergence of corporate social responsibility in Chile: the importance of authenticity and social networks. J. Bus. Ethics 86, 191–206. doi: 10.1007/s10551-009-0190-1

[ref8] Bentler P. M. (1990). Comparative fit indexes in structural models. Psychol. Bull. 107, 238–246. doi: 10.1037/0033-2909.107.2.238, PMID: 2320703

[ref9] Brammer S. Millington A. Rayton B. (2007). The contribution of corporate social responsibility to organizational commitment. Int. J. Hum. Resour. Manage. 18, 1701–1719. doi: 10.1080/09585190701570866

[ref10] Brown R. (1958). How shall a thing be called? Psychol. Rev. 65, 14–21. doi: 10.1037/h0041727, PMID: 13505978

[ref11] Browne M. W. Cudeck R. (1993). “Alternative ways of assessing model fit” in Testing Structural Equation Models. eds. Bollen K. A. Long J. S. (Newbury Park, CA: Sage), 136–162.

[ref12] Bryk A. S. Raudenbush S. W. (1992). Hierarchical Linear Models: Applications and Data Analysis Methods. Thousand Oaks, CA: Sage Publications, Inc.

[ref13] Burgoon E. M. Henderson M. D. Markman A. B. (2013). There are many ways to see the forest for the trees: a tour guide for abstraction. Pers. Psychol. Sci. 8, 501–520. doi: 10.1177/1745691613497964, PMID: 26173209

[ref14] Carroll G. R. Wheaton D. R. (2009). The organizational construction of authenticity: an examination of contemporary food and dining in the U.S. Res. Organ. Behav. 29, 255–282. doi: 10.1016/j.riob.2009.06.003

[ref15] Chen Y. R. Hung-baesecke C. F. (2014). Examining the internal aspect of corporate social responsibility (CSR): leader behavior and employee CSR participation. Commun. Res. Repor. 31, 210–220. doi: 10.1080/08824096.2014.907148

[ref16] Davies G. Chun R. da Silva R. V. Roper S.. (2001). The personification metaphor as a measurement approach for corporate reputation. Corp. Repu. Rev. 4, 113–127. doi: 10.1057/palgrave.crr.1540137

[ref17] De Roeck K. Maon F. (2018). Building the theoretical puzzle of employees’ reactions to corporate social responsibility: an integrative conceptual framework and research agenda. J. Bus. Ethics 149, 609–625. doi: 10.1007/s10551-016-3081-2

[ref18] De Roeck K. Marique G. Stinglhamber F. Swaen V. (2014). Understanding employees’ responses to corporate social responsibility: mediating roles of overall justice and organisational identification. Int. J. Hum. Resour. Manage. 25, 91–112. doi: 10.1080/09585192.2013.781528

[ref19] Du S. Bhattacharya C. B. Sen S. (2011). Corporate social responsibility and competitive advantage: overcoming the trust barrier. Manag. Sci. 57, 1528–1545. doi: 10.1287/mnsc.1110.1403

[ref20] Evans W. R. Davis W. D. (2011). An examination of perceived corporate citizenship, job applicant attraction, and CSR work role definition. Bus. Soc. 50, 456–480. doi: 10.1177/0007650308323517

[ref21] Finegan J. E. (2000). The impact of person and organizational values on organizational commitment. J. Occup. Organ. Psychol. 73, 149–169. doi: 10.1348/096317900166958, PMID: 34648546

[ref22] Gilbert D. Malone P. (1995). The correspondence bias. Psychol. Bull. 117, 21–38. 7870861 10.1037/0033-2909.117.1.21

[ref23] Glavas A. Piderit S. K. (2009). How does doing good matter? J. Corp. Citizen. 2009, 51–70. doi: 10.9774/GLEAF.4700.2009.wi.00007

[ref01] Gond J.-P. El-Akremi A. Igalens J. Swaen V. (2010). Corporate social responsibility influence on employees. Int. Center Corp. Social Respons. 54, 1–47.

[ref24] Gond J.-P. El Akremi A. Swaen V. Babu N. (2017). The psychological microfoundations of corporate social responsibility: a person-centric systematic review. J. Organ. Behav. 38, 225–246. doi: 10.1002/job.2170

[ref25] Grant A. M. (2007). Relational job design and the motivation to make a prosocial difference. Acad. Manag. Rev. 32, 393–417. doi: 10.5465/amr.2007.24351328

[ref26] Hameed I. Riaz Z. Arain G. A. Farooq O. (2016). How do internal and external CSR affect employees’ organizational identification? A perspective from the group engagement model. Front. Psychol. 7:788. doi: 10.3389/fpsyg.2016.00788, PMID: 27303345 PMC4884747

[ref27] Hawn O. Ioannou I. (2016). Mind the gap: the interplay between external and internal actions in the case of corporate social responsibility. Strateg. Manage. J. 37, 2569–2588. doi: 10.1002/smj.2464

[ref28] Hofman P. S. Newman A. (2014). The impact of perceived corporate social responsibility on organizational commitment and the moderating role of collectivism and masculinity: evidence from China. Int. J. Hum. Resour. Manage. 25, 631–652. doi: 10.1080/09585192.2013.792861

[ref29] Hox J. J. (1995). Applied Multilevel Analysis. Amsterdam: TT-publikaties.

[ref30] Hox J. J. Maas C. J. M. (2001). The accuracy of multilevel structural equation modeling with pseudo balanced groups and small samples. Struct. Equat. Model. Multidiscip. J. 8, 157–174. doi: 10.1207/S15328007SEM0802_1

[ref31] Hu L. Bentler P. M. (1999). Cutoff criteria for fit indexes in covariance structure analysis: conventional criteria versus new alternatives. Struct. Equat. Model. Multidiscip.J. 6, 1–55. doi: 10.1080/10705519909540118

[ref32] Ionescu L. (2021). Corporate environmental performance, climate change mitigation, and green innovation behavior in sustainable finance. Econo. Manage. Fin. Markets 16, 94–106. doi: 10.22381/emfm16320216

[ref33] Jones D. A. (2010). Does serving the community also serve the company? Using organizational identification and social exchange theories to understand employee responses to a volunteerism programme. J. Occup. Organ. Psychol. 83, 857–878. doi: 10.1348/096317909X477495

[ref34] Kang Sangjin. (2016). Multilevel Models. Seoul: Hakjisa.

[ref35] Kivetz Y. Tyler T. R. (2007). Tomorrow I’ll be me: the effect of time perspective on the activation of idealistic versus pragmatic selves. Organ. Behav. Hum. Decis. Process. 102, 193–211. doi: 10.1016/j.obhdp.2006.07.002

[ref36] Klein K. J. Kozlowski S. W. J. (eds) (2000). Multilevel Theory, Research, and Methods in Organizations: Foundations, Extensions, and New Directions. San Francisco, CA: Jossey-Bass.

[ref37] Kline R. B. (2011). “Convergence of structural equation modeling and multilevel modeling,” in The SAGE Handbook of Innovation in Social Research Methods. eds. Williams M. Vogt W. P. (London: Sage Publications Ltd.), 562–589.

[ref38] Lammers J. (2012). Abstraction increases hypocrisy. J. Exp. Soc. Psychol. 48, 475–480. doi: 10.1016/j.jesp.2011.07.006

[ref39] Lange D. Washburn N. T. (2012). Understanding attributions of corporate social irresponsibility. Acad. Manag. Rev. 37, 300–326. doi: 10.5465/amr.2010.0522

[ref40] Maas C. J. M. Hox J. J. (2004). Robustness issues in multilevel regression analysis. Statistica Neerlandica 58, 127–137. doi: 10.1046/j.0039-0402.2003.00252.x, PMID: 35448984

[ref41] Maas C. J. M. Hox J. J. (2005). Sufficient sample sizes for multilevel modeling. Methodology 1, 86–92. doi: 10.1027/1614-2241.1.3.86, PMID: 34276510

[ref42] May A. Y. C. Hao G. S. Carter S. (2021). Intertwining corporate social responsibility, employee green behavior, and environmental sustainability: the mediation effect of organizational trust and organizational identity. Econo. Manage. Fin. Markets 16, 32. doi: 10.22381/emfm16220212

[ref43] McNeish D. M. Stapleton L. M. (2016). The effect of small sample size on two-level model estimates: a review and illustration. Educ. Psychol. Rev. 28, 295–314. doi: 10.1007/s10648-014-9287-x

[ref44] McShane L. Cunningham P. (2012). To thine own self be true? Employees’ judgments of the authenticity of their organization’s corporate social responsibility program. J. Busin. Ethics 108, 81–100. doi: 10.1007/s10551-011-1064-x

[ref45] McWilliams A. Siegel D. S. (2000). Corporate social responsibility and financial performance: correlation or misspecification? Strateg. Manage. J. 21, 603–609. doi: 10.1002/(SICI)1097-0266(200005)21:5<603::AID-SMJ101>3.0.CO;2-3, PMID: 35792853

[ref46] Meuleman B. Billiet J. (2009). A Monte Carlo sample size study: how many countries are needed for accurate multilevel SEM? Survey Res. Methods 3, 45–58. doi: 10.18148/srm/2009.v3i1.666

[ref47] Meyer J. P. Allen N. J. (1991). A three-component conceptualization of organizational commitment. Hum. Resour. Manage. Rev. 1, 61–89. doi: 10.1016/1053-4822(91)90011-z

[ref48] Mowday R. T. Steers R. M. Porter L. W. (1979). The masurement of organizational commitment: a progress report. J. Vocat. Behav. 14, 224–247. doi: 10.1016/0001-8791(79)90072-1

[ref49] Muthén B. O. (1991). Multilevel factor analysis of class and student achievement components. J. Edu. Meas. 28, 338–354. doi: 10.1111/j.1745-3984.1991.tb00363.x

[ref50] Muthén B. O. (1994). Multilevel covariance structure analysis. Sociologi. Methods Res. 22, 376–398. doi: 10.1177/0049124194022003006, PMID: 35611602

[ref51] Nunnally J. C. Bernstein I. H. (1994). Psychometric Theory. New York, NY: McGraw-Hill.

[ref52] Orlitzky M. Benjamin J. D. (2001). Corporate social performance and firm risk: a meta-analytic review. Bus. Soc. 40, 369–396. doi: 10.1177/000765030104000402

[ref53] Orlitzky M. Schmidt F. L. Rynes S. L. (2003). Corporate social and financial performance: a meta-analysis. Organ. Studies 24, 403–441. doi: 10.1177/0170840603024003910, PMID: 26086454

[ref54] Preacher K. J. Zyphur M. J. Zhang Z. (2010). A general multilevel SEM framework for assessing multilevel mediation. Psychol. Methods 15, 209–233. doi: 10.1037/a0020141, PMID: 20822249

[ref55] Raudenbush S. W. Bryk A. S. (2002). Hierarchical Linear Models: Applications and Data Analysis Methods. Thousand Oaks, CA: Sage.

[ref56] Rupp D. E. Mallory D. B. (2015). Corporate social responsibility: psychological, person-centric, and progressing. Annu. Rev. Organ. Psychol. Organ. Behav. 2, 211–236. doi: 10.1146/annurev-orgpsych-032414-111505

[ref57] Rupp D. E. Ganapathi J. Aguilera R. V. Williams C. A. (2006). Employee reactions to corporate social responsibility: an organizational justice framework. J. Organ. Behav. 27, 537–543. doi: 10.1002/job.380

[ref58] Rupp D. E. Shao R. Skarlicki D. P. Paddock E. L. Kim T. Y. Nadisic T. (2018). Corporate social responsibility and employee engagement: the moderating role of CSR-specific relative autonomy and individualism. J. Organ. Behav. 39, 559–579. doi: 10.1002/job.2282

[ref59] Ryu E. (2014). Model fit evaluation in multilevel structural equation models. Front. Psychol. 5, 1–9. doi: 10.3389/fpsyg.2014.00081 24550882 PMC3913991

[ref60] Schaefer A. D. Pettijohn C. E. (2006). The relevance of authenticity in personal selling: is genuineness an asset or liability? J. Mark. Theory Pract. 14, 25–35. doi: 10.2753/mtp1069-6679140102

[ref61] Sen S. Bhattacharya C. B. Korschun D. (2006). The role of corporate social responsibility in strengthening multiple stakeholder relationships: a field experiment. J. Acad. Mark. Sci. 34, 158–166. doi: 10.1177/0092070305284978

[ref62] Snijders T. A. B. Bosker R. J. (1993). Standard errors and sample sizes for two-level research. J. Educ. Stat. 18, 237–259. doi: 10.3102/10769986018003237, PMID: 28301201

[ref63] Stites J. P. Michael J. H. (2011). Organizational commitment in manufacturing employees: relationships with corporate social performance. Bus. Soc. 50, 50–70. doi: 10.1177/0007650310394311

[ref64] Suh Y. J. (2016). The role of relational social capital and communication in the relationship between CSR and employee attitudes: A multilevel analysis. J. Leader. Organ. Stud. 23, 410–423. doi: 10.1177/1548051816637564

[ref65] Trope Y. Liberman N. (2010). Construal level theory of psychological distance. Psychol. Rev. 117, 440–463. doi: 10.1037/a0018963, PMID: 20438233 PMC3152826

[ref66] Turker D. (2009). Measuring corporate social responsibility: a scale development study. J. Bus. Ethics 85, 411–427. doi: 10.1007/s10551-008-9780-6, PMID: 30337901

[ref67] Valentine S. Fleischman G. (2008). Ethics programs, perceived corporate social responsibility and job satisfaction. J. Bus. Ethics 77, 159–172. doi: 10.1007/s10551-006-9306-z

[ref68] Vătămănescu E.-M. Dabija D. C. Gazzola P. Cegarro-Navarro J. G. Buzzi T. (2021). Before and after the outbreak of Covid-19: linking fashion companies’ corporate social responsibility approach to consumers’ demand for sustainable products. J. Clean Prod. 321:128945. doi: 10.1016/j.jclepro.2021.128945

[ref69] Vermeulen F. Barkema H. (2002). Pace, rhythm, and scope: process dependence in building a profitable multinational corporation. Strateg. Manage. J. 23, 637–653. doi: 10.1002/smj.243

[ref70] Vlachos P. A. Panagopoulos N. G. Rapp A. A. (2013). Feeling good by doing good: employee CSR-induced attributions, job satisfaction, and the role of charismatic leadership. J. Bus. Ethics 118, 577–588. doi: 10.1007/s10551-012-1590-1

[ref71] Vlachos P. A. Panagopoulos N. G. Bachrach D. G. Morgeson F. P. (2017). The effects of managerial and employee attributions for corporate social responsibility initiatives. J. Organ. Behav. 38, 1111–1129. doi: 10.1002/job.2189

[ref72] Waddock S. A. Graves S. B. (1997). The corporate social performance-financial performance link. Strateg. Manage. J. 18, 303–319. doi: 10.1002/(SICI)1097-0266(199704)18:4<303::AID-SMJ869>3.0.CO;2-G

